# Association of handgrip strength and CT-derived body composition with insulin resistance in women with prediabetes and newly diagnosed type 2 diabetes

**DOI:** 10.20945/2359-4292-2026-0056

**Published:** 2026-06-08

**Authors:** Elham Yousief, Mona Khozam, Ghada Ayeldeen, Mohamed Hamadna Allah El Ghobashy

**Affiliations:** 1 Department of Internal Medicine, Faculty of Medicine, Cairo University, Cairo, Egypt

**Keywords:** Keywords:, Handgrip strength, insulin resistance, HOMA-IR, visceral adiposity, CT imaging, prediabetes, type 2 diabetes, women

## Abstract

**Objective:**

To examine the associations of handgrip strength and CT-derived muscle and visceral fat indices with insulin resistance in middle-aged women with prediabetes and newly diagnosed type 2 diabetes.

**Subjects and methods:**

This cross-sectional study included 44 women aged 40-60 years attending a tertiary endocrinology clinic. Participants were classified according to the American Diabetes Association (ADA, 2022) criteria as having prediabetes (n = 29) or newly diagnosed type 2 diabetes (n = 15). All participants underwent anthropometric assessment, biochemical testing, L3-level CT imaging for visceral adipose tissue (VAT) and psoas muscle measurements, and handgrip strength evaluation using a digital dynamometer. Insulin resistance was assessed using the homeostatic model assessment for insulin resistance (HOMA-IR). Multivariable linear regression analysis was performed to identify independent factors associated with HOMA-IR.

**Results:**

Dominant-hand grip strength demonstrated an inverse association with HOMA-IR (β = -0.297, p = 0.033). VAT volume and BMI were not significantly associated with HOMA-IR after adjustment (p > 0.75). The overall model explained 28% of the variance in HOMA-IR (R^2^ = 0.28; adjusted R^2^ = 0.14). Psoas muscle thickness showed a borderline inverse relationship with HOMA-IR (p = 0.079).

**Conclusion:**

In this cohort of women with early glucose dysregulation, handgrip strength was independently associated with insulin resistance, whereas visceral adiposity was not. These findings suggest that muscle performance may represent a clinically relevant correlation of early metabolic impairment. Longitudinal studies are needed to clarify the temporal relationships.

## INTRODUCTION

Prediabetes and type 2 diabetes mellitus (T2DM) are increasingly prevalent among middle-aged women and are strongly influenced by alterations in body composition. The accumulation of visceral adipose tissue has traditionally been considered a principal contributor to insulin resistance because of its endocrine activity and inflammatory signaling properties (^1^-^3^). However, skeletal muscle is the primary site of insulin-mediated glucose disposal, accounting for about 70%-80% of postprandial glucose uptake. Consequently, changes in muscle quantity or functional capacity may significantly influence insulin sensitivity independently of adiposity (^4^,^5^).

Recent studies suggest that muscle strength may provide clinically relevant information beyond measures of muscle mass alone. Functional decline can precede measurable reductions in cross-sectional muscle area, reflecting early impairments in contractile efficiency and metabolic flexibility (^6^-^8^). In women, particularly during midlife, shifts in fat distribution and muscle quality may further modify metabolic risk profiles (^9^,^10^). Prospective data from Asian populations demonstrate that lower muscle strength is independently associated with an increased incidence of prediabetes and T2DM after adjustment for visceral adiposity (^1^). Similarly, cross-sectional analyses have reported that reduced muscle performance correlates with markers of insulin resistance across diverse cohorts (^11^,^12^).

Although visceral adiposity is consistently associated with cardiometabolic disease, its independent contribution to insulin resistance seems to vary according to disease stage and population characteristics (^13^-^15^). Some investigations suggest that combined muscle-fat indices may better reflect metabolic health than fat measures alone (^2^,^16^,^17^). Furthermore, interventional studies demonstrate that resistance training improves insulin sensitivity even in the absence of significant reductions in fat mass, underscoring the metabolic importance of muscle performance (^18^-^20^).

Despite growing interest in this field, the relative associations of functional muscle strength and CT-derived visceral adiposity with insulin resistance remain insufficiently characterized in women with early glucose abnormalities. Therefore, this study aims to evaluate the relationships between handgrip strength, CT-based body composition parameters, and insulin resistance measured by HOMA-IR in women with prediabetes and newly diagnosed T2DM.

## SUBJECTS AND METHODS

### Study design and setting

A cross-sectional observational study was conducted at the Kasr El Ainy Centre for Endocrinology and Diabetes, Faculty of Medicine, Cairo University, from August 2022 to December 2023. The objective is to examine the associations between skeletal muscle performance, CT-derived body composition parameters, and insulin resistance among women with early disturbances in glucose metabolism.

Given the cross-sectional design, analyses were restricted to evaluating statistical associations without inferring temporal direction or causality.

### Participants

Women aged 40-60 years attending the outpatient endocrinology clinic were consecutively screened for eligibility. Participants were included if they met the American Diabetes Association (ADA, 2022) criteria for either prediabetes or newly diagnosed type 2 diabetes mellitus (T2DM).

Prediabetes was defined as: Fasting plasma glucose between 100-125 mg/dL and/or; HbA1c between 5.7%-6.4%.

Newly diagnosed T2DM was defined as: fasting plasma glucose ≥ 126 mg/dL and/or; HbA1c ≥ 6.5%, in individuals who had not initiated glucose-lowering therapy.

Exclusion criteria included any medical condition or pharmacologic treatment known to significantly influence muscle mass, fat distribution, or insulin sensitivity. These included thyroid dysfunction, Cushing’s syndrome, chronic liver disease, chronic kidney disease, malignancy, polycystic ovary syndrome, and current use of corticosteroids, insulin sensitizers, or anabolic agents.

A total of 44 eligible participants were enrolled. Sample size estimation was based on detecting moderate correlations (r ≥ 0.35) with 80% statistical power at a 5% significance level.

### Ethical approval

The study protocol was reviewed and approved by the Research Ethics Committee of the Faculty of Medicine, Cairo University (Approval No. MD-322-2022). All participants provided written informed consent prior to enrollment. The study adhered to the principles of the Declaration of Helsinki.

### Clinical and anthropometric assessment

Standardized anthropometric measurements were obtained. Body weight and height were measured with participants wearing light clothing and no footwear. Body mass index (BMI) was calculated as weight (kg) divided by height squared (m^2^). Waist circumference was measured at the midpoint between the lower costal margin and the iliac crest.

These conventional indices were included to enable comparison between anthropometric markers of adiposity and imaging-derived measurements.

### Laboratory evaluation

Venous blood samples were collected following an overnight fast of at least eight hours. Laboratory analyses included: fasting plasma glucose; fasting insulin; HbA1c; lipid profile (total cholesterol, triglycerides, HDL-C, LDL-C); alanine aminotransferase (ALT); aspartate aminotransferase (AST).

Insulin resistance was estimated using the homeostasis model assessment formula:

HOMA-IR = (Fasting glucose × Fasting insulin) / 405.

HOMA-IR was analyzed as a continuous variable and served as the primary metabolic outcome.

### CT-Based body composition assessment

Non-contrast computed tomography imaging was performed at the level of the third lumbar vertebra (L3) using a 64-slice multidetector scanner. The L3 region was selected due to its established correlation with total-body skeletal muscle and visceral fat compartments.

Using dedicated image-analysis software, the following parameters were quantified: visceral adipose tissue (VAT) volume (cm^3^); Psoas muscle index (PMI), calculated as total psoas cross-sectional area normalized to height (cm^2^/m^2^); Psoas muscle thickness (PMTH), calculated as the mean of bilateral measurements.

These imaging-derived metrics were used to characterize structural muscle and fat compartments.

### Muscle strength measurement

Muscle performance was assessed using a calibrated digital hand dynamometer. Participants were positioned according to standardized procedures and instructed to perform three maximal voluntary contractions with each hand, with one-minute rest intervals between attempts.

Hand dominance was self-reported. The mean value of the dominant hand was used for statistical analysis to ensure methodological consistency across participants.

Reduced muscle strength was defined according to the European Working Group on Sarcopenia in Older People (EWGSOP2) criteria.

### Statistical analysis

Statistical analyses were performed using SPSS version 28.0 (IBM Corp., Armonk, NY, USA).

Continuous variables were assessed for normality using the D’Agostino-Pearson test. Normally distributed variables are shown as mean ± standard deviation, while skewed data are expressed as median with interquartile range.

Associations between HOMA-IR and body composition variables were evaluated using Pearson or Spearman correlation coefficients, as appropriate.

To identify variables independently associated with insulin resistance, multivariable linear regression analysis was conducted with HOMA-IR as the dependent variable. Independent variables entered into the model included: dominant-hand grip strength; visceral adipose tissue volume; Psoas muscle index; Psoas muscle thickness; BMI; age.

Model assumptions, including linearity and absence of multicollinearity, were verified prior to interpretation. Results are reported as standardized beta coefficients (β) with corresponding p-values. A two-sided p-value < 0.05 was considered statistically significant.

### Methodological considerations

The cross-sectional design limits interpretation to associations. Menopausal status, physical activity level, dietary intake, and inflammatory biomarkers were not systematically assessed and may represent potential confounders. These factors are acknowledged as limitations and should be addressed in future longitudinal studies.

## RESULTS

### Participant characteristics

The study included 44 women aged between 40 and 60 years, with a mean age of 45.5 ± 5.9 years. According to the American Diabetes Association (ADA, 2022) criteria, 29 participants (65.9%) met the definition of prediabetes, while 15 (34.1%) were classified as having newly diagnosed type 2 diabetes. **Table 1** one summarizes the baseline demographic, metabolic, and body composition parameters.

**Table 1:** Baseline characteristics of participants

Parameter	Mean ± SD/Median (IQR)
Age (years)	45.5 ± 5.9
BMI (kg/m^2^)	34.6 ± 6.8
Waist circumference (cm)	98.2 ± 10.3
Fasting glucose (mg/dL)	112.4 ± 41.5
Fasting insulin (µIU/mL)	13.3 ± 6.6
HOMA-IR	3.72 ± 2.25
QUICKI	0.32 ± 0.03
Triglycerides (mg/dL)	125.0 (86.5-188.5)
Visceral adipose tissue (cm^3^)	295.5 (150.3-436.8)
Handgrip strength (right, kg)	17.5 ± 5.4
Handgrip strength (left, kg)	16.8 ± 5.2
PMI (cm^2^/m^2^)	7.45 ± 2.74
PMTH (cm)	6.23 ± 0.71

The cohort demonstrated elevated adiposity overall, with a mean BMI of 34.6 ± 6.8 kg/m^2^ and a median visceral adipose tissue (VAT) volume of 295.5 cm^3^ (interquartile range: 150.3-436.8). Mean dominant-hand grip strength was 17.5 ± 5.4 kg. Structural muscle parameters showed a mean psoas muscle index (PMI) of 7.45 ± 2.74 cm^2^/m^2^ and a mean psoas muscle thickness (PMTH) of 6.23 ± 0.71 cm. The mean HOMA-IR value was 3.72 ± 2.25, reflecting variability in insulin resistance across participants.

### Bivariate associations

Correlation analyses were conducted to explore the relationships between insulin resistance and body composition measures (**Table 2**).

**Table 2:** Correlation coefficients between muscle and metabolic parameters

Parameter	HOMA-IR (r)	p-value
Fasting insulin	0.78	< 0.001
Fasting glucose	0.64	< 0.001
Handgrip strength (right)	-0.29	0.033
Handgrip strength (left)	-0.19	0.07
Visceral adipose tissue volume	0.04	0.81
PMTH	-0.18	0.08
BMI	0.02	0.75

HOMA-IR showed strong positive correlations with fasting insulin (r = 0.78, p < 0.001) and fasting plasma glucose (r = 0.64, p < 0.001), supporting the internal consistency of metabolic measurements.

**Figure 1:**
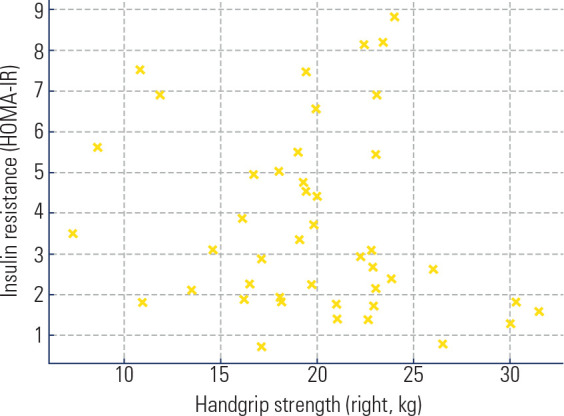
Inverse association between handgrip strength and insulin resistance (HOMA-IR).

An inverse correlation was observed between dominant-hand grip strength and HOMA-IR (r = -0.29, p = 0.033). Participants with lower grip strength tended to exhibit higher levels of insulin resistance. **Figure 1** illustrates this relationship, where the fitted regression line shows a negative slope.

Scatter plot illustrating the inverse relationship between dominant-hand grip strength and insulin resistance (HOMA-IR). The regression line demonstrates a modest negative association (r = -0.29, p = 0.033).

**Figure 2:**
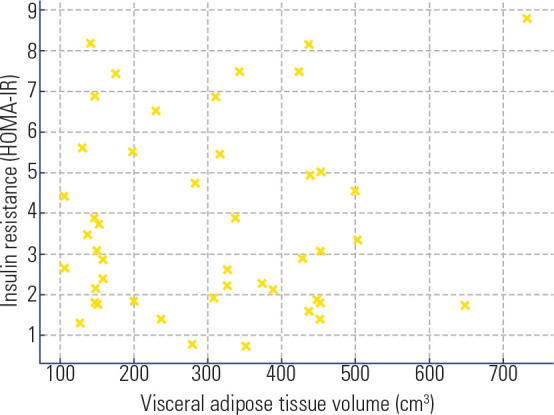
Relationship between visceral fat volume and insulin resistance.

In contrast, VAT volume did not demonstrate a statistically significant correlation with HOMA-IR (r = 0.04, p = 0.81). **Figure 2** shows the absence of a clear linear pattern. Similarly, BMI showed no meaningful association with insulin resistance (r = 0.02, p = 0.75).

Scatter plot showing the relationship between visceral adipose tissue (VAT) volume and insulin resistance (HOMA-IR). No significant linear association was observed (r = 0.04, p = 0.81).

PMTH displayed a weak inverse relationship with HOMA-IR (r = -0.18, p = 0.08), while PMI was not significantly correlated.

### Multivariable regression analysis

To examine independent associations, multivariable linear regression analysis was performed with HOMA-IR as the dependent variable (**Table 3**).

**Table 3:** Multiple linear regression predicting HOMA-IR

Predictor	β Coefficient	p-value	Interpretation
Handgrip strength (right)	-0.297	0.033	Significant inverse predictor
Handgrip strength (left)	0.251	0.067	Trend toward significance
PMTH	-0.784	0.079	Suggestive inverse relationship
PMI	0.259	0.163	Not significant
Visceral adipose tissue volume	0.0006	0.815	No predictive effect
BMI	0.018	0.754	No predictive effect
Age	-0.027	0.687	No predictive effect

After adjustment for age, BMI, VAT volume, PMI, and PMTH, dominant-hand grip strength remained independently associated with HOMA-IR (β = -0.297, p = 0.033). The negative beta coefficient indicates that lower muscle strength corresponded to higher levels of insulin resistance.

Neither VAT volume (β = 0.0006, p = 0.815) nor BMI (p = 0.754) retained statistical significance in the adjusted model. PMI also did not demonstrate an independent association (p = 0.163).

**Figure 3:**
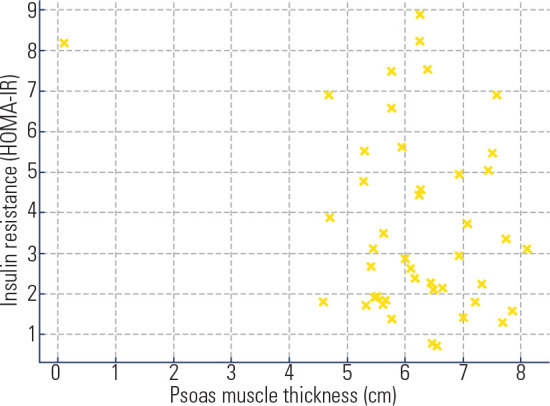
Relationship between muscle thickness and metabolic risk

PMTH showed a borderline inverse association (β = -0.784, p = 0.079), suggesting a possible relationship that warrants further investigation. **Figure 3** shows the distribution of PMTH in relation to HOMA-IR.

Scatter plot depicting the relationship between psoas muscle thickness (PMTH) and insulin resistance (HOMA-IR). A modest inverse trend was observed (β = -0.784, p = 0.079).

The final regression model explained 28% of the observed variability in HOMA-IR (R^2^ = 0.28; adjusted R^2^ = 0.14), indicating that additional unmeasured factors likely contribute to insulin resistance in this population.

### Summary of observed associations

Within this cohort of women with early glucose abnormalities: Dominant-hand grip strength was independently associated with insulin resistance. Visceral adipose tissue volume was not independently associated with HOMA-IR. Structural muscle thickness demonstrated a borderline inverse relationship.

These findings describe cross-sectional associations and should not be interpreted as indicating temporal or causal relationships.

## DISCUSSION

This study examined the relationships between muscle performance, imaging-derived body composition parameters, and insulin resistance in women with prediabetes and newly diagnosed type 2 diabetes. The primary observation was that dominant-hand grip strength was independently associated with HOMA-IR, whereas visceral adipose tissue volume was not statistically significant after adjustment for covariates. These findings describe associative patterns within this cohort and should be interpreted accordingly.

### Interpretation in the context of previous research

Skeletal muscle plays a central role in glucose disposal under insulin-stimulated conditions. Functional impairment of muscle may therefore influence systemic glucose regulation even in the absence of marked structural atrophy. Several observational studies have reported inverse relationships between muscle strength and markers of glucose dysregulation. For example, longitudinal analyses in Asian populations have demonstrated that individuals with lower baseline muscle strength were more likely to develop prediabetes or type 2 diabetes during follow-up (^1^). Similarly, cross-sectional investigations have linked reduced muscle performance with higher insulin resistance indices across diverse adult populations (^11^,^18^).

Importantly, growing evidence suggests that muscle strength and muscle mass may not convey identical metabolic information. Some studies have indicated that functional measures such as handgrip strength show stronger correlations with metabolic outcomes than imaging-based muscle area alone (^6^,^17^). This distinction aligns with the current findings, in which dominant-hand grip strength remained associated with HOMA-IR, while psoas muscle index did not demonstrate independent significance.

With respect to adiposity, visceral fat has traditionally been considered a principal contributor to insulin resistance because of its pro-inflammatory and endocrine properties (^2^,^3^). However, imaging-based studies have reported variable associations between visceral fat volume and insulin resistance once muscle-related parameters are included in multivariable models (^13^,^15^). In this analysis, visceral adipose tissue volume was not independently associated with HOMA-IR after adjustment. This does not negate the metabolic relevance of adiposity but suggests that, within this sample, muscle performance demonstrated a stronger statistical relationship with insulin resistance than VAT volume.

The borderline association observed between psoas muscle thickness and HOMA-IR further supports the notion that muscle characteristics may relate to metabolic function. Although this finding did not reach conventional statistical significance, the direction of association was consistent with prior reports describing inverse relationships between muscle quality markers and insulin resistance (^5^,^14^). Larger studies would be necessary to clarify this relationship.

### Clinical considerations

Handgrip strength is a simple and reproducible measure that can be obtained in routine clinical settings without specialized imaging. While CT-derived metrics provide precise quantification of body compartments, they are less feasible for broad metabolic screening. The association between grip strength and HOMA-IR suggests that functional muscle assessment may contribute additional information when evaluating metabolic status among women with early glucose abnormalities. However, these findings should be interpreted as exploratory and hypothesis-generating.

### Limitations

Several limitations warrant consideration.

First, the cross-sectional design precludes inference regarding temporal sequence or causality. It cannot be determined whether reduced muscle strength contributes to insulin resistance or reflects metabolic impairment.

Second, the sample size was modest, which may have limited statistical power to detect smaller associations, particularly for structural muscle measures.

Third, menopausal status was not systematically documented. Given the age range of participants, hormonal transitions may have influenced both body composition and insulin sensitivity. This variable should be incorporated into future investigations.

Fourth, physical activity level, dietary intake, and inflammatory markers were not assessed and may represent unmeasured confounders.

Finally, CT analysis was based on a single L3 slice, which, although validated, may not fully capture whole-body fat and muscle distribution.

In conclusion, in this cohort of women with prediabetes and newly diagnosed type 2 diabetes, dominant-hand grip strength was independently associated with insulin resistance as measured by HOMA-IR, whereas visceral adipose tissue volume was not statistically significant in adjusted analyses. These findings highlight the potential relevance of muscle performance as a correlate of metabolic dysfunction in early-stage glucose abnormalities.

Given the observational nature of the study, prospective research is required to determine whether changes in muscle strength precede, accompany, or follow alterations in insulin sensitivity. Future studies incorporating larger samples and longitudinal designs will be essential to clarify the clinical implications of these associations.

## Data Availability

datasets related to this article will be available upon request to the corresponding author.

## Availability of data and materials:

the datasets used and/or analyzed during this study are available from the corresponding author upon reasonable request. Raw CT imaging data were handled in accordance with institutional privacy and ethical guidelines.
